# Per- and Polyfluoroalkyl Substances (PFAS) in Integrated Crop–Livestock Systems: Environmental Exposure and Human Health Risks

**DOI:** 10.3390/ijerph182312550

**Published:** 2021-11-28

**Authors:** Gaurav Jha, Vanaja Kankarla, Everald McLennon, Suman Pal, Debjani Sihi, Biswanath Dari, Dawson Diaz, Mallika Nocco

**Affiliations:** 1 Department of Land, Air and Water Resources, University of California, Davis, CA 95616, USA; imddiaz@ucdavis.edu (D.D.); manocco@ucdavis.edu (M.N.); 2 Department of Soil and Crop Sciences, Texas A&M University, College Station, TX 77843, USA; vkankarla@tamu.edu; 3 Department of Crop and Soil Science, Oregon State University, Klamath Falls, OR 97603, USA; everald.mclennon@oregonstate.edu; 4 Department of Internal Medicine, University of New Mexico Health Sciences Center, Albuquerque, NM 87106, USA; spal@salud.unm.edu; 5 Department of Environmental Sciences, Emory University, Atlanta, GA 30322, USA; debjani.sihi@emory.edu; 6 Agricultural and Natural Resources, Cooperative Extension at North Carolina Agricultural and Technical State University, Greensboro, NC 27411, USA; bdari@ncat.edu

**Keywords:** forever chemicals, livestock contaminations, chronic kidney disease, remediation, environmental justice, groundwater contaminants, exposure pathway, foaming agent, renal dysfunction

## Abstract

Per- and polyfluoroalkyl substances (PFAS) are highly persistent synthetic organic contaminants that can cause serious human health concerns such as obesity, liver damage, kidney cancer, hypertension, immunotoxicity and other human health issues. Integrated crop–livestock systems combine agricultural crop production with milk and/or meat production and processing. Key sources of PFAS in these systems include firefighting foams near military bases, wastewater sludge and industrial discharge. Per- and polyfluoroalkyl substances regularly move from soils to nearby surface water and/or groundwater because of their high mobility and persistence. Irrigating crops or managing livestock for milk and meat production using adjacent waters can be detrimental to human health. The presence of PFAS in both groundwater and milk have been reported in dairy production states (e.g., Wisconsin and New Mexico) across the United States. Although there is a limit of 70 parts per trillion of PFAS in drinking water by the U.S. EPA, there are not yet regional screening guidelines for conducting risk assessments of livestock watering as well as the soil and plant matrix. This systematic review includes (i) the sources, impacts and challenges of PFAS in integrated crop–livestock systems, (ii) safety measures and protocols for sampling soil, water and plants for determining PFAS concentration in exposed integrated crop–livestock systems and (iii) the assessment, measurement and evaluation of human health risks related to PFAS exposure.

## 1. Introduction

The burgeoning global population has increased the need for maximizing food production while simultaneously minimizing its ecological footprint. Climate change challenges demand resilient, sustainable and economically competitive agricultural practices such as integrated crop–livestock systems. Integrated crop–livestock systems (ICLS) have played an important role in enhancing practices to improve soil health, nutrient cycling, pathological management, weed infestation and optimizing the nutrient losses from the system. We define ICLS as a combined system including plant and livestock components that have synergistic impacts on agricultural, economic and environmental outcomes. Here, we discuss ICLS in the context of environmental exposure pathways for contaminant mobility from sources such as soil and groundwater, which eventually affect crop biomass, livestock and dairy products, as well as human health. Managing and regulating contaminants in ICLS are a major challenge. In developed countries such as the United States, federal regulations, such as the National Pollutant Discharge Elimination System (NPDES), and state regulatory orders, keep track of exposure pathways in ICLS and ensure they remain below the allowable screening limits for environmental and human exposure. These screening and regulatory limits are defined for commonly occurring contaminants such as nitrate and metal(loid)s; however, the risk and extent of impacts from emerging contaminants such as per- and polyfluoroalkyl substance (PFAS) compounds are unknown and still require extensive research. Research and decisions regarding PFAS currently focus on regulatory studies for drinking water and food products. This paper highlights PFAS exposure in the environment as a holistic pathway affecting soils, water, plants, livestock and dairy products.

Per- and polyfluoroalkyl substance compounds are among the most pervasive environmental contaminants which bio accumulate, move long distances in the environment and persist through the food chain [1,2,3]. They are a class of fluorinated synthetic compounds with unique multiple carbon–fluorine bonds, which are highly polar and display amphiphilic properties [4]. Approximately 4700 of these compounds were developed in the late 1940s and are used in various industrial and commercial processes. Because of their strong carbon chain, in addition to fluorine atoms, they do not degrade easily and remain in the environment for a long time. Therefore, it is difficult to estimate the environmental half-life of these compounds. They originate from regular use and disposal of consumer products such as non-stick cookware, clothes and carpets resistant to stains and the formulation of aqueous fire-fighting foams. Fire-fighting foams are retardants used for fires and a variety of other industries, including aerospace, automotive, construction, electronics and military. In agroecosystems, abiotic or biotic degradation of PFAS release various other impurities into the environment commonly containing perfluorooctane sulfonic acid (PFOS), perfluorooctanoic acid (PFOAs), perfluoro hexane sulfonic acid (PFHxS), perfluorononanoic acid (PFNA), Perfluorodecanoic acid (PFDeA), and many more [5,6].

**Figure 1:**
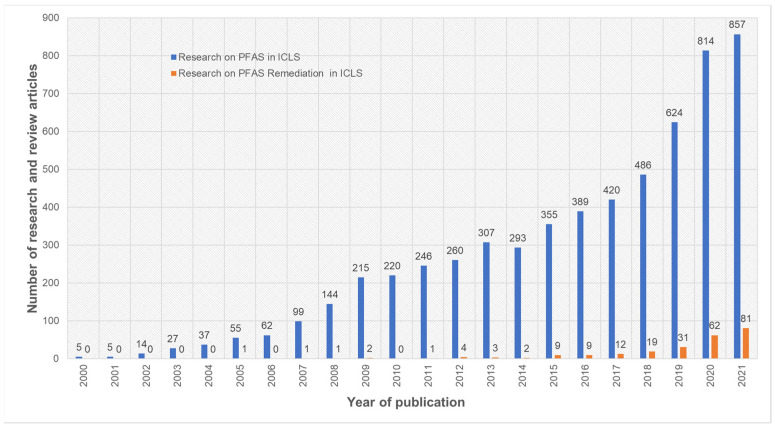
Number of research and review articles published on PFAS in integrated crop–livestock agroecosystem (ICLS) research over last decades (between 2000 to 2021). The plot was generated using Web of Science search engine using keywords with Boolean operations as (PFAS OR PFOS OR PFOA OR PFBA OR PFBS OR PFPeA OR PFHxS OR PFHxA OR GenX OR PFHpA OR PFNA) AND (Environment OR Human exposure OR Livestock OR Soil OR Milk OR Crop OR Dairy) AND (Remediation).

PFOS and PFOA are the contaminants commonly studied for their long-term persistence, and they are commonly detected in wastewater treatment, fresh water and ground water systems during environmental risk analysis [7,8,9,10,11,12]. Their sources of origin (Table 1) include industrial emissions, consumer products, contaminated drinking water, surface water and house dust [11,12]. We conducted a systematic analysis of published research and review articles over the last two decades (2000–2021) on 14 November 2021, using the Web of Science search engine. Our analysis indicated that the number of research articles published on PFAS in ICLS increased over the last two decades (Figure 1). More specifically, there was roughly a four-fold increase in PFAS research work in ICLS over the last decade. Moreover, we observed an increasing trend in published work on PFAS remediation in ICLS over the last nine years (2012–2021) based on our search criteria. However, the articles published on PFAS remediation in ICLS were limited in both spatial and temporal scales to laboratory experiments or other emerging techniques. Therefore, this emerging need for PFAS assessment and remediation in ICLS warrants an extensive review and discussion.

## 2. “Forever Chemicals”: Persistence and Mobility

Anthropogenic activities are widely regarded as the major source of PFAS contamination. The sources of PFAS in the environment depend on production methods or application during the manufacturing of other products [26]. Primary manufacturing industries produce PFAS and secondary production facilities use PFAS to produce goods for industrial, commercial and consumer applications [27]. Point source facilities include industrial and military training sites as well as wastewater treatment plants [16]. Industries of the military, textiles, leather and paper products often require PFAS in either training programs (military) or in production processes. As a result, PFAS are often present in surface water and groundwater near industrial, military and wastewater treatment sites. Aqueous Film Forming Foam (AFFF) is a major compound in combating fires and its effectiveness as a fire retardant is often attributed to the presence of PFAS [28]. Other sources of PFAS in agriculture include sewage sludge, bio-solids and treated wastewater, which become inadvertent point sources for PFAS contamination even when judiciously used [29]. Ubiquitous in the environment, PFAS are found in air, water, soil, vegetation and livestock, and thus can be threat to human health if not properly regulated and managed.

Acquiring the epithet “forever chemicals” is not a coincidence. Per- and polyfluoroalkyl substances are tremendously resistant to biological and chemical degradation [30]. The persistence of PFAS in the environment is strongly associated with the length and strength of carbon fluorine chain structures where strength increases with increasing chain lengths [30]. Additionally, PFAS with five or more carbon atoms tend to have higher octanol-water partitioning coefficients than short chain PFAS compounds, which make short chain PFAS compounds more soluble than their long chain counterparts [23]. A negative consequence of the unique physical and chemical properties is the provision of several pathways of PFAS to have long-term stability in soils. Additionally, being biotically stable, they are impervious to both metabolic and bacterial breakdown [31]. Since PFAS are resistant to biotic degradation, once in water or soil, humans and wildlife can have direct or indirect contact through their diets from bioaccumulation in the food chain. In fact, PFAS were added to the Persistent Organic Pollutant (POPs) list under the Stockholm Convention in 2009 for their persistence and subsequent negative environmental and health impacts [32]. There is evidence of biological degradation of perfluorinated chains by breaking bonds one carbon at a time starting at the carboxyl end, with carbon dioxide and fluoride as end products [33]. There is research underway explaining the biological and enzymatic degradation of the “forever chemicals”. Destructive methods can also be based on photolysis, enzymatic reduction or supercritical water oxidation. The bacterial degradation of PFOA and PFOS compounds by *Acidimicrobium* sp. led to the buildup of fluoride, short-chain perfluorinated products and acetate in pure and enriched culture using ammonium or hydrogen as the electron donor [34]. In addition to microbial degradation, the carbon–fluorine bond cleavage is also documented due to reductive defluorination by an organohalide-respiring microbial community [35].

PFAS contamination in soil has received much attention because of the role soil structure plays in PFAS chemical and physical transformation, as well as PFAS movement within or between environmental media. The protein component of soil organic matter (SOM) serves as an excellent sorption site for PFAS making SOM a significant PFAS reservoir [36]. PFAS persistence in soil is also attributed to its ability to partition in soils [37]. Brusseau et al. referred to soils as a long-term source of contamination because SOM serves as a sink to collect and store PFAS from industrial sources as well as a conduit to transfer PFAS to drinking water sources (rivers, lakes, surface water and groundwater) [38]. This leads to a cyclical exposure pathway of contaminations across soil, biota and the atmosphere [25]. Once in soils, PFAS compounds are soluble and can be transported into surface water or leach into groundwater, which leads to bioaccumulation in plants and animals, especially in ICLS. There is a high potential for PFAS uptake by forages and tubers [39]. Additionally, PFAS can be transferred from soil to plant roots via diffusion and sorption [40]. Based on a plethora of research data, it is evident that the occurrence of PFAS concentration in soils is a global issue as distribution of PFAS were more prominent in soils when compared with other media such as air, surface water, or groundwater [25]. Moreover, it was observed that PFAS not only occur at varying levels in both urban and rural areas, but also among diverse soil types over prolonged periods [41,42,43,44,45]. Total continental PFSAs concentration ranged between 29–14,300 and 7–3270 pg/g for PFCA and PFSA, respectively [46].

The physical and chemical behavior of PFAS in soils has been linked to specific PFAS manufacturing and production processes. One type of PFAS production process results in a molecular structure with significant side chains or branching, whereas another produces linear molecules with little or no branching. For example, Washington et al., in a study in Decatur, Alabama, USA, observed the persistence of PFAS in sludge-applied soils correlated to the length of the carbon–fluorine bonds—longer chained bonds resulted in less mobility, and vice-versa [47]. On contrary, polluted groundwater was associated with shorter chained (<8) PFAS carbon–fluorine bonds [31]. The production processes affect the surface-active behavior, and determine if PFAS compounds are either hydrophobic, oleophobic, lipophobic, or hydrophilic [48]. Uncertainty and complexity of PFAS behavior can be explained in part by the hydrophobic backbone, polar or ionic head structure and functional group of the head structure. Both the hydrophobic C-F backbone and the hydrophilic functional head can control sorption/solvation in environment [49].

## 3. Human Health Impacts and Exposure

In our exploration of PFAS impacts on ICLS, it is crucial to consider how PFAS exposure can adversely impact human health. The changing composition of PFAS during use, cumulative nature of bioaccumulation, potential for delayed and/or long-term health effects, as well as the modification of effects by coexisting environmental factors make it challenging to study the health effects of PFAS. However, several studies have shown an association with various adverse health issues in humans with exposure to PFAS. Effects on renal health, endocrine function, metabolism and bone health have been well studied and reported [50,51,52,53,54,55,56,57,58,59,60]. The health effects of intrauterine exposure to PFAS have also been investigated [50,61,62,63,64].

A growing body of literature suggests that PFAS exposure disrupts endocrine function and metabolism. Dysregulation of thyroid hormones have been studied most frequently. Exposure to PFAS has been positively associated with free thyroxine levels, though there has been no demonstrated association with Thyroid Stimulating Hormone levels [51]. The association varies with different PFAS, gender, iodine levels and smoking status [52]. Changes in glucose and lipid metabolism are also noted with PFAS exposure [53,54]. The prevalence of metabolic syndrome and its individual components have been found to have an association with PFAS exposure though the results are only consistent across studies for PFNA (perfluorononanoic acid) [53]. An effect on glucose metabolism may be particularly relevant in pregnancy, where PFOS exposure has a positive association with increased glucose levels, although overt gestational diabetes mellitus has not been seen [54].

Adverse kidney health has also been linked with exposure to different types of PFAS. Kidneys are the major route of elimination for PFAS, particularly short carbon chain, carboxylic acid functional group or branched isomer forms [55]. Three studies based on the National Health and Nutrition Examination Survey cohort found an association between direct PFOA and PFOS exposure, renal dysfunction (decreased Estimated Glomerular Filtration Rate) and prevalence of chronic kidney diseases among both adults and children [55]. Another cohort study noted an inverse relationship between serum perfluoro hexane sulfonic acid (PFHxS), perfluorononanoic acid (PFNA), perfluorodecanoic acid (PFDeA) levels and Estimated Glomerular Filtration Rate [56]. The effects of PFAS exposure in renal health may go beyond chronic kidney disease. An association has been postulated with renal and genitourinary cancers, though the data remain sparse and large-scale cohort studies with longer follow-up are required to further elucidate the role [55,57].

Toxicological studies have demonstrated several cellular and metabolic derangements associated with short- and long-term PFAS exposure, which provide insight into possible mechanisms. In animal models, exposure to PFOS in particular has been shown to cause cell death by inducing enzymes of cell apoptosis such as capsases and cytochrome c, and reduce antioxidant enzymes leading to oxidative stress [55,65]. This alteration in nuclear transcription may be mediated by the dysregulation of PPAR (peroxisome proliferators-activated receptors) pathways, which are involved modulation of gene expression in cells [55]. Enhanced endothelial permeability through actin filament remodeling has also been demonstrated with PFAS exposure, which could be a key mechanism of podocyte injury leading to chronic kidney disease [66].

Prenatal exposure to PFAS has been associated with reduced bone mass in young females and was attenuated when adjusted to body composition [50]. In addition, early childhood exposure has also been associated with lower bone mineral density (BMD) with a possible response related to the intensity and/or magnitude of exposure [58]. Repeated exposure to PFAS is also associated with worse bone health [59]. In a small sample of young male subjects, PFAS exposure was also associated with increase in osteoporosis risk and risk of fracture [60]. A mechanism suggested has been the affinity of PFAS to hydroxyapatite in bones [60]. Endocrine modifying effects have also been suggested to play a role [59]. The findings of the effects of PFAS on skeletal health in early life are particularly important as childhood and youth are important determinants of fracture risk later in life [58].

Different PFAS have been found in maternal blood and cord blood, indicating placental transmission [67,68,69]. Maternal exposure to PFAS is widespread, with detectable levels found in serum in 98% of studied pregnant women in one study [61]. Exposure to higher levels of PFOA and PFHpS (Perfluoroheptanesulfonic acid) have been found to be associated with higher odds of miscarriage [62]. Perfluorononanoic acid (PFNA) exposure has been linked to risk of preterm birth [61]. Prenatal PFAS exposure may also be associated with both maternal and fetal thyroid dysfunction though the effect seemed to vary with individual compounds [63]. A large epidemiological study in Italy also noted higher rates of severe small-for-gestational-age (SGA) births among populations living in PFAS-contaminated regions, though no link with SGA was found with maternal PFAS exposure in a small cohort of pregnant women in USA [61,64].

There is increasing recognition that human health does not exist in a void, but is interconnected with the health of the shared environment with soil, plants, water and livestock [70]. A ‘One Health’ approach involves combining the institutional knowledge of professions in these interconnected fields [70]. This is particularly relevant to PFAS, where a variety of human activities lead to PFAS generation, environmental contamination, exposure to ICLS and human exposure pathways simultaneously occurring in close geographical proximity.

## 4. Fate of PFAS Compounds in Integrated Crop–Livestock Systems

**Figure 2:**
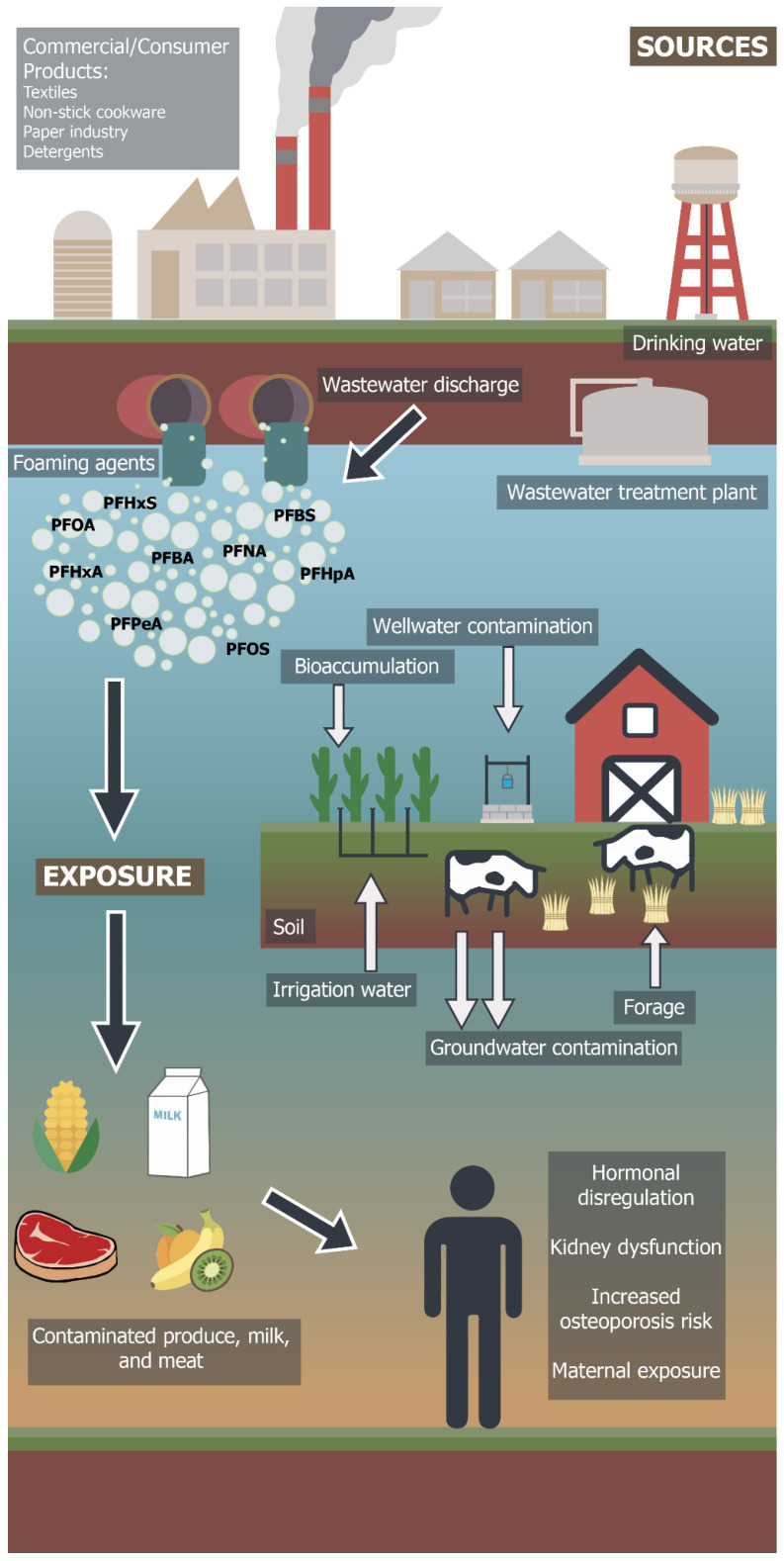
Potential human exposure pathway of PFAS contaminants from commercial/industrial sources through crop livestock agroecosystems (Artwork by Dawson Diaz).

The PFAS group of chemicals cycle through water, soil, crops, dairy and meat products in ICLS and impact human health through various exposure pathways (Figure 2). Diet and drinking water are the main routes of human exposure to PFAS [71]. The presence of PFAS has been reported in the surface/subsurface water and groundwater of leading dairy producing countries such as India, the United States and China, including drinking water supplies [72,73]. According to research based on a recursive regression model of India’s ICLS, groundwater use accounts for 38% of the total value of milk output, whereas surface water use accounts for 15% of the same output [74]. However, water from the Ganges river of India has detectable concentrations of PFAS chemicals, mainly PFOA and PFOS [75,76]. The flux estimates of PFAS chemicals from Ganges River is reported to be in the range of several hundreds of kilograms per year [77]. Although these chemicals are reported below international standards, their persistence poses danger for biomagnification and accumulation at higher trophic levels in harmful concentrations, specifically for cattle and crop production.

As of January 2021 in the United States, 2337 locations from 49 states were known to have PFAS contamination in water samples from military sites [78]. Most humans in the United States are exposed to PFAS, with blood, serum and urine containing PFOA and PFOS compounds [79]. In 2017, a well water district was shut down in Maine after PFAS were identified in the water samples, which also lead to the shutdown of a nearby ICLS with detectable levels of PFAS in soils, hay, milk and human blood samples [80]. The United States Food and Drug Administration has reported levels of PFAS in milk samples analyzed from one of the New Mexico dairies possessing potential human health concerns [81]. The PFAS compounds found in the milk samples from this dairy were PFOA (47-169 parts per trillion) and PFOS (881–5680 parts per trillion) [81]. Approximately 5000 cattle consumed PFAS-contaminated groundwater in this dairy [81]. The contamination emerged from a foaming agent used in the nearby air force base [81]. The Food Safety and Inspection Service of the United States Department of Agriculture ceased dairy production and shipment of cattle from the contaminated NM dairy until a baseline analysis and further research data on PFAS depletion kinetics are produced [82]. Well samples monitored in Madison, Wisconsin (the second largest dairy producing state in the United States), reported the presence of 18 different types of PFAS compounds across domestic, municipal and agricultural wells, ranging from 2.5 to 47 parts per trillion [83].

The PFAS contamination emerges from industrial wastes, contaminated sludges or foaming agents and infiltrates into the soil matrix, surface water and groundwater. Groundwater that is used for irrigating forage crops in ICLS can integrate PFAS into the leaf tissue through bioaccumulation [84]. Feeding hay to cattle contaminates dairy products that are consumed by humans [85]. PFAS can be associated with microplastics in lake environments and PFAS sorption onto microplastics can be enhanced in the presence of organic matter [86]. Once incorporated into food webs, PFAS compounds move across different trophic levels of food webs accumulating in different concentration ranges. Table 2 provides the examples of studies that reported different levels of PFAS compounds (mainly PFOS and PFOA) in different components of ICLS. Bioaccumulation of PFAS is related to the concentration and type of PFAS in cattle feed or forage. Long chain PFAS (10–20 fluorinated carbons) compounds ingested through livestock feed or watering have lower potential for removal from urine or milk and higher potential for accumulation and biomagnification in beef/tissue of cattle than short chain (nine fluorinated carbons) compounds [87,88,89]. The estimated half-life of PFOS in dairy cows is approximately 56 days based on a pharmacokinetic model describing the uptake of contamination by cows through contaminated forage vs. its elimination from milk [89]. However, this conclusion was based on assumption of complete elimination of consumed contamination through milk samples [90]. More research on accumulation and biomagnification factors in meat is required for developing regulatory policies for different groups of PFAS compounds based on its half-life, retention and assimilation in cattle.

## 5. Limited Global Regulations and Standards to Address Environmental Concerns

Toxicological impacts of PFAS are site-specific, and regulations depend on socioeconomic and political factors [25]. The U.S. EPA has developed sampling methods for potable and non-potable water. However, there are no published protocols for analyzing bioaccumulated PFAS in forage crops or soil samples. There are currently no residential or regional screening levels available through federal guidelines for PFAS compounds in soils in the United States for agricultural or industrial limits. Agricultural, industrial and commercial soil screening levels for different PFAS compounds have been identified and defined by Health Canada [98]. For example; soil screening values for PFOA and PFOS in agricultural soils are 0.70 and 2.1, respectively [98]. In 2009, the United Nations Environment Programme’s Stockholm Convention listed PFOA (salts and related compounds) and PFOS in Annex A and C [99,100,101]. Annex A aims at *eliminating* the production and use whereas Annex C aims at *restricting* the production and use of chemicals [99,100,101]. The current health advisory levels of PFOA and PFOS from drinking water developed by the U.S. EPA have been established at a lifetime exposure of 70 parts per trillion [102]. However, it is important to develop limits, regulatory guidelines and screening levels for livestock watering to reduce the accumulation and transport of PFAS compounds in milk, meat and other dairy products to eliminate pathways to human exposure.

Investigations of PFOS, PFOA and PFBS in the United States take place under the Comprehensive, Environmental Response, Compensation and Liability Act, Toxic Substances Control Act, the Safe Drinking Water Act and the Defense Environmental Restoration Program. The Compensation and Liability Act follows investigation of PFOA and PFOS under EPA’s residential screening levels for tap water (0.4 ppb at hazard quotient = 1) and soil (1.3 ppm at hazard quotient = 1). The research underway by the U.S. EPA has validated test methods for 29 PFAS compounds, facilitated the clean-up of contaminated groundwater sites, developed the significant new use rule to regulate manufacture and import of PFOA and PFOS and provided assistance to more than 30 states for developing new tools for research and communications [103]. Research findings to date have focused widely on the need for regulation standards in drinking water; however, it is important to expand monitoring and develop regulatory standards for milk, meat and agricultural commodities. The sampling methods that are currently used for soil, potable and non-potable water, milk and human serum are listed in Table 3. Various agencies have developed unique sampling methods (Table 3) from simple liquid chromatography to isotope dilution to carefully separate out contaminants. These technologies are continually advancing to achieve higher efficiency.

Several cases of milk contaminants have been reported in the US states such as Maine, New Mexico, Wisconsin, Alabama, but to date, no guideline values have been developed by regulatory agencies. In 2017, the Maine Center for Disease Control developed an action level for PFOS in cow’s milk of 210 ppb after contaminated samples were found in hay, monitoring well samples, milk and groundwater used for drinking and livestock watering [104]. Maine CDC developed this action level based on the U.S. EPA’s recommendations of a reference dose of 20 ng/kg of body weight per day for PFOS in drinking water [105]. Maine Department of Environmental Protection has also developed remedial action guidelines for soil in crop-based dairy systems considering a soil-hay-cow-milk exposure pathway model of 1,700,000 ng/kg dry weight [105]. The remedial action guidelines are based on an EPA reference dose for drinking water that uses an incidental soil ingestion exposure pathway for children [105]. The current discrepancies and knowledge gaps in formulating the action and guideline values require more research on residual toxicity in environment and biomagnification on different trophic levels.

The European Commission severely restricted the use of PFOS in Europe, except for the space industry. However, in 2020 the EU banned the production and marketing of PFOA salts and precursors for all industrial uses including space exploration [106]. The PFAS contaminants (PFOA, PFOS, PFNA and PFHxS) are now restricted under regulatory framework on food contaminants by the European Union [107]. The German Ministry of Health proposed regulatory guidelines of maximum 300 parts per trillion of combined PFOA and PFOS for human health exposure. 

## 6. Remediation and Preventive Strategies

Soil and water are precursors to plant and animal food chains. The use of PFAS-contaminated soil and water can lead to entry of PFAS compounds into leaf tissues (forage, pasture or vegetables/fruits), edible produce, meat and dairy products. These chemicals cycle through water, soil, crop, dairy and meat products in an integrated livestock system and impact the human health. Assessing the risks and levels of PFAS in soils and water could help prevent the bioaccumulation in crops and livestock used for human consumption. Therefore, most of the remediation and preventive techniques presented here are more focused on soil and water. These measures are intended to prevent the migration of PFAS compounds into crops and livestock products by keeping the levels of these contaminants below the guideline values/residential screening levels.

In addition to conventional remediation techniques, new techniques are constantly being developed for PFAS remediation in soils and water. Most of the previous techniques, such as incineration and adsorption using activated carbon, have known challenges such as cost, energy consumption, difficulty with disposal and extreme operating conditions [108,109]. Many passive approaches have had partial success such as adsorption, filtration, reverse osmosis, enhanced photolysis, electrochemical oxidation and sonochemical destruction.

Conventional soil PFAS remediation methods include soil washing, excavation and thermal oxidation, chemical oxidation, ball milling, electron beams, immobilization methods, excavation and landfilling (Table 3). Some of the most effective PFAS remediation strategies known as ‘treatment train processes’ have been in use for in situ remediation which uses multiple synergistic technologies [110]. Some partially effective treatment methods were anion exchange, granular activated carbon (GAC) and reverse osmosis [111].

The manipulation of materials and structures at nanoscale dimensions, i.e., nanotechnology, has recently been used as one of the most innovative and promising technologies for PFAS remediation. As an example, engineered nanomaterials use a large specific surface area and the quantum nature of energy states to have more accessible adsorption sites and higher surface reactivities [112,113]. This enables effective remediation of contaminated water by adsorbing the PFAS compounds in the exposed surfaces of the nanomaterials [112,113,114]. Another technique with great potential for replication to remove PFAS is the use of modified nanosized iron oxides with high adsorption capacity and magnetic properties which serve as ideal sorbents for PFAS under multiple conditions [115]. Similarly, nano-photocatalysts under UV irradiation can be used to decompose PFOA in the presence of TiO_2_-based, Ga_2_O_3_-based, or In_2_O_3_ catalysts [115]. Research on remediation of PFAS in water is underway and evolving every day. Techniques for PFAS remediation include physical, chemical and biological approaches integrated within ICLS. These remediation techniques can be classified into three major categories: (a) soil and sediment remediation, (b) water remediation and (c) biological remediation.

### 6.1. Soil and Sediment Remediation Methods

Soil remediation techniques are based on adsorption/separation behavior, soil washing and thermal treatment, soil liquefaction, excavation and placement in impermeable materials or offsite disposal in landfills, sonochemical destruction, advanced oxidation or reduction processes, ball milling or vapor generation methods [116,117]. Soil adsorption/separation methods commonly use soil stabilization [116], where the PFAS sorbents are added and mixed with soil to stabilize and immobilize PFAS compounds [117]. In general, the sorbent adsorbs PFAS and reduces the potential of PFAS leaching to the groundwater. However, this approach works best in soil compared with water because PFAS release tends to occur on the surface, and some PFAS chemicals have a strong affinity to soil [16]. This method has significant importance in managing PFAS contamination in military bases. Alternatively, powdered or granular activated carbon (e.g., carbon fibers, BioNuchar etc.) with a porous structure and strong heterogeneous surface is used to sorb and remove harmful compounds [118,119,120]. Soil washing involves the separation of contaminants from soil and sediments through the application of water, solvents or air bubbles on the contaminated soil or water [121]. Thermal treatment involves increasing the soil temperature to 500–600 °C to vaporize organic contaminants and break PFAS compounds in the gas stream at a temperature of 1200 °C [122,123]. Another useful technique named soil liquefaction uses foam fractionation techniques to detach PFAS from liquefied soil and water to the induced bubbles [124].

In recent years, other in situ and off-site ways to remediate PFAS have been developed. One such technique is excavation, which can be used in two different ways. First, excavating and placing soils in impermeable materials reduces infiltration, isolates impacted material and controls seepage by a proper drainage system. It is a short-term, uncertain solution. Often, contamination may still be present on-site, and any small physical disturbance can spread the contaminants. Thus, the impermeable materials require continuous monitoring and long-term management [125,126]. On the contrary, excavation and offsite disposal in landfills involves reduction in the volume and concentration of PFAS and immobilization before locating in an offsite landfill. However, this process needs additional steps to collect, destroy and monitor all PFAS separately, and should not be considered as a preferred remediation technique from a management standpoint [127,128].

Ex situ methods such as sorption, filtration and sonochemical destruction methods help lower toxicity by degradation of PFOS and PFOA in soil and water [129]. One such technique includes sonolysis, which uses sound waves at 20–1100 kHz frequencies to facilitate cavitation in water and create bubbles with large surface area enabling decomposition of PFAS [130,131]. In the advanced oxidation/reduction process, contaminants (phenols, endocrine-disrupting chemicals) are destroyed either by direct anodic oxidation or in solution using strong oxidants generated by cathodic electrochemical reactions [132,133]. In general, this process is versatile, has a long life span, is energy efficient, automated and cost-effective [134]. In addition, ozonation is a commonly used advanced oxidation process for water treatment plants in the United States [135]. In recent times, use of an electron beam is considered as a more advanced oxidation-reduction process, involving irradiating material with accelerated electrons to destroy PFAS. However, this electron beam approach has been used only on wastewater and aqueous solutions, thus warrants further testing for its applicability to ICLS [136,137]. Another common method similar to ball milling is a mechanochemical (MC) destruction method, which employs mechanical force such as shaking to allow reactions on the surface of ball mills to effectively destroy PFOS and PFOA [138]. In addition, vapor energy generators use 1100 °C steam to destroy PFAS from impacted soils in an ex situ treatment chamber [139].

### 6.2. Water Remediation Methods

The principal techniques in remediation of water from PFAS include adsorption and separation using foam fractionation, reverse osmosis or nanofiltration, resin membrane-based ion exchange, incineration, electrochemical oxidation (ozonation and ozofractionation), photolysis, enzymatic reduction and water oxidation. In adsorption and separation, PFAS adsorbent materials are introduced into the contaminated waters to immobilize PFAS using adsorption and separation techniques. This method can also use foam fractionation to detach PFAS from liquefied soil and water with induced bubbles [124]. The advanced oxidation process of PFAS contaminant removal can be destructive by direct anodic oxidation—contaminants (phenols, endocrine-disrupting chemicals) adsorb onto the anode surface and are destroyed by an electron transfer reaction or oxidation reaction through strong oxidants generated by cathodic electrochemical reactions [135]. It is an evolving destructive technology for many PFAS. Ozonation is a commonly used advanced oxidation process method for water treatment plants in the United States. This method treats liquid waste by chemically oxidizing organic contaminants and forming concentrated foam fractionates, which can be separated from the treated water [140,141].

There are some economically feasible and environmentally sustainable water remediation methods which have been developed in recent years. For example, photolysis in aqueous solutions involves producing products such as carbonates and bicarbonate radicals which react to produce PFAS molecules with shorter chains [142]. Another technique called supercritical water oxidation uses the super critical state of water (water at temperature 373.9 °C and pressure of 221.1 bar), where the chemical oxidation process breaks down strong carbon fluorine bonds and decomposes various hazardous organic pollutants into non-toxic steam waste [143,144,145].

### 6.3. Microbial and Phytoremediation

Phytoremediation could be an effective approach for controlling PFAS, as with many other contaminants of concern in the environment. Limited information on plant accumulation and analytical techniques for bioavailable fractions in plant roots have restricted these remediation methods. However, research and protocols for monitoring PFAS exposure pathways have initiated some concepts and principles to apply in remediating the contaminants from rhizosphere using degradation techniques. Some of these techniques include rhizodegradation, phytoextraction, phytotransfer, mycoremediation and biodegradation. Rhizodegradation is a plant-assisted bioremediation in which organisms residing in the root zone alter or degrade chemicals [146]. It is an aerobic process facilitated by enzyme catalyzed (oxidative) degradation [147]. Rhizodegradation is cost efficient, low maintenance and most sustainable for PFAS-contaminated sites; however, the process can be a slow and long-term approach [148]. Some phytoremediation candidate species include (a) the wetland species *Juncus effuses*, that has been demonstrated to accumulate 11.4% of seven PFAS compounds from PFAS-spiked soil [146], and (b) *Betula pendula* and *Picea abies*, which were reported to accumulate up to 97 and 94 ng g^−1^ of PFAS compounds at a firefighting training site near Stockholm, Sweden [147]. The PFAS contaminants can also be degraded by lignolytic fungi or via biodegradation by aerobic bacteria into non-toxic compounds such as carbon dioxide, water and ammonia [149,150]. The ability of Gram-positive and Gram-negative bacteria to break down PFAS depends on soil organic matter content, and involves electrostatic and hydrostatic connections in the bacterial sorption of compounds [151].

## 7. Exposure and Equity

Human exposure to PFAS has been a public health concern due to its high environmental persistence. Research on location or clusters of certain industries in low income, underserved communities and related adverse health impacts has expanded significantly over recent years, raising issues of environmental justice [152,153]. PFAS contamination is also an environmental justice problem. Accumulating scientific evidence has linked adverse outcomes in community health of low-income households and underserved communities inhabiting within five miles of PFAS sites [154,155]. A comparison of over 70 non-military sites found that 39,000 more low-income households and 295,000 more underserved people were within five miles of sites contaminated with PFAS [156]. In the US state of Michigan, environmental inequities are even more pronounced, with 36,170 more low-income households and 134,488 more underserved people living within five miles of a PFAS-contaminated site [156].

Public policy initiatives have been increasingly investigating environmental quality as an indicator of individual human health and well-being [157]. As environmental risks are not evenly distributed across socioeconomic groups, additional considerations for exposure assessment have included socioeconomic factors of affected populations [158]. Framed as environmental justice or the inequitable distribution of risks and exposure, numerous studies have reported detrimental environment effects that have disproportionately affected socially disadvantaged and underserved populations. Whereas a myriad of factors affect health outcomes, socioeconomic status and race have been found to highly correlate with health and environmental inequalities showing strong associations and translating inequalities to inequities [159,160]. Studies have also shown that socioeconomic factors influence contaminant exposure and specific health outcomes [158]. Disparities in human exposure to harmful chemicals such as PFAS have also been shown to exist among educational statuses. For example, underserved communities with lower levels of educational attainment and political capital often have less access to funding to conduct hazard identification, exposure analysis and health related effects [161]. Moreover, it was observed that poor access to health care information and healthcare means lower health promotion rates, lower risk avoidance, a less healthy diet and more adverse conditions that increase susceptibility to exposure [162]. Underserved communities are underrepresented in media and underserved by government entities [163]. Other factors include polluting industries or those with the greatest risk to produce harmful chemicals and pollute the environment are more likely to be located near low-income communities. These groups are often placed at a disproportionately higher risk for environmental chemical exposure [164]. Therefore, accounting for institutional differences and understanding actions by government entities in perpetuating resource injustice, is a prerequisite for advancing equitable solutions related to PFAS contamination and exposure [165].

In our systematic review, we did not find any studies examining the impact of PFAS exposure related to soil, plants, livestock, and water adjacent to underserved communities. Moreover, we did not find any studies examining the feasibility and any additional challenges for PFAS remediation in socially and economically disadvantaged agricultural communities. This is an important area for future interdisciplinary work in order to avoid further inequities from arising related to exposure, remediation and monitoring.

## 8. Conclusions

In integrated crop–livestock systems, PFAS compounds are impacting surface water and groundwater by infiltrating through soils from industrial sources of contamination. Once groundwater is contaminated, it can lead to exposure pathways of bioaccumulation in plants and cattle contaminating the entirety of farm produce and dairy products. Consumption of these contaminated products leads to severe human health issues. There is evidence of PFAS contamination in milk and meat samples from dairies in countries such as the United States and China. Therefore, more vigorous research and regulatory guidelines are critical and required for monitoring and developing screening guidelines not only for the dairy products but also for soil, groundwater, forage and crops. Lack of assessment of the extent of exposure pathways in underserved communities living near PFAS sites may be further advancing disparate adverse health impacts. However, more research is required to understand the combined agronomic and epidemiological impacts for developing diet regulatory guidelines.

## Figures and Tables

**Table 1:** Summary of major point and nonpoint production and manufacturing sources of PFAS released to the environment.

Industry/Source	PFAS Compound(s)	Uses	Reference
Textile, electrical, metal, laundry and cleaning industries	PFOA, PFOS, PFBA and other PFAS	Industrial, commercial and consumer products	[13,14]
Aqueous Film Forming Foams (AFFFs)	PFOA, PFOS, PFBA and other PFAS	Fire training facilities/airports, military bases	[15,16,17]
Landfill leachate/waste disposal	PFOA, PFOS, PFBA, PFHxS and other PFAS	Reservoir for products containing PFAS chemicals that undergo decomposition, disposal of waste during primary and secondary manufacturing process using PFAS	[15,18,19]
Printing/paper product production	PFOA, PFOS, PFBA and other PFAS	Surface coatings to repel grease and moisture	[13,20]
Wastewater treatment plants/biosolids, recycled water	PFOA, PFOS, PFBA and other PFAS	Application of treated wastewater especially in agricultural lands water from manufacturing, industrial and household wastewater which are sources of PFOA and PFOS	[15,17,21,22,23,24]
Commercial and industrial products	PFOA, PFOS, PFBA and other PFAS	Products that repel water and oil in the textiles, paper industries (paper and packaging, clothing, carpets, nonstick cookware, pharmaceutical and personal care products (cosmetics, toothpaste), agricultural products (pesticides, herbicides), industrial (wire coating and insulation, corrosion prevention, surfactant, fluoroplastics, fluoropolymers, rubber)	[14,16,25]

**Table 2:** Examples of reported levels of PFOS and PFAS in individual components of ICLS.

Component	Description	PFOS	PFOA	Source
Soil	Includes agricultural, background and secondary-source contaminated soils	3–5,500,000 ng/kg	10–2,531,000 ng/kg	[38,91]
Water	Rainwater and groundwater used for irrigation and or livestock	0.073–113 ng/L	23–2752 ng/L	[89,91,92]
Milk	Includes raw, retail and full-cream milk	n.d.–9060 ng/L	n.d.–151.8 ng/L	[88,89,93,94,95]
Meat	Beef Muscle Beef Liver	21–2700 ng/kg 24–91,000 ng/kg	7–500 ng/kg 9–114,000 ng/kg	[89,96] [89,96,97]
Crops	Cereal grains (silage, wheat, barely, maize)	3.9–860 ng/kg	8.3–39,300 ng/kg	[89,91]

**Table 3:** Sampling methodology and storage techniques for PFAS contaminants in soil, water, milk, meat and human serum.

Matrix	Method Name	Developed by	Technology or Instrumentation	Sampling and Storage Methods	Remarks
Soil	ASTM D7968 17a	ASTM International	Liquid Chromatography/Tandem Mass Spectrometry (LC/MS/MS)	Sampled in polypropylene containers 2 g soil required per analysis Sample should be shipped on ice below 6 degrees Analysis should be completed in 28 days	This method is applicable to determine 21 PFAS compounds
Non-potable water	SW-846 Method 8327	USEPA	Multiple Reaction Monitoring (MRM) Liquid Chromatography/Tandem Mass Spectrometry (LC/MS/MS)	Samples should be collected in HDPE containers. Samples should be immediately freezed below 6 degrees Celsius until analyzed Non-formal holding time by USEPA is 28 days	This method measures 24 PFAS compounds. Matrix-Groundwater, surface water and wastewater
	SW-846 Method	USEPA	Isotope Dilution Method	Research underway	Collaborative efforts of USEPA and Department of Defense to analyze non-drinking water, biosolids and sediments
Potable or drinking water	Method 537.1	USEPA	Solid Phase Extraction and Liquid Chromatography/Tandem Mass Spectrometry (LC/MS/MS)	Samples should be collected in polypropylene bottles 5 g/L Preservation reagent “Trizma” should be added to each sampling container Sample extraction should take place within 14 days of sampling	This method is applicable to determine 18 PFAS compounds
Milk	C-010.01	USFDA	Liquid Chromatography/Tandem Mass Spectrometry (LC/MS/MS); modified QuEChERS extraction technique	Collected in 500 mL LDPE bottles Preserved below −20 degrees Celsius until analyzed	This method is applicable to determine 16 PFAS compounds and can be used for milk, bread, lettuce and fish as matrices.
Meat (beef)		USDA	Methanolic Extraction analyzed by Liquid Chromatography/Tandem Mass Spectrometry	Meat and plasma samples collected at slaughter or processing industry. Approximately 5 g of sample collected in homogenized tube mill and ground at 5000 rpm for 2 min.	It is applicable to bovine muscle and plasma and can be analysed for 16 PFAS compounds

## Data Availability

Not applicable.
